# Comprehensive genomic characterization and expression analysis of calreticulin gene family in tomato

**DOI:** 10.3389/fpls.2024.1397765

**Published:** 2024-04-22

**Authors:** Tayeb Muhammad, Tao Yang, Baike Wang, Haitao Yang, Diliaremu Tuerdiyusufu, Juan Wang, Qinghui Yu

**Affiliations:** ^1^ Key Laboratory of Genome Research and Genetic Improvement of Xinjiang Characteristic Fruits and Vegetables, Institute of Horticulture Crops, Xinjiang Academy of Agricultural Sciences, Urumqi, China; ^2^ College of Computer and Information Engineering, Xinjiang Agricultural University, Urumqi, China

**Keywords:** tomato, CRT gene family, endoplasmic reticulum, bioinformatics, abiotic stress, gene expression

## Abstract

Calreticulin (CRT) is a calcium-binding endoplasmic reticulum (ER) protein that has been identified for multiple cellular processes, including protein folding, regulation of gene expression, calcium (Ca^2+^) storage and signaling, regeneration, and stress responses. However, the lack of information about this protein family in tomato species highlights the importance of functional characterization. In the current study, 21 CRTs were identified in four tomato species using the most recent genomic data and performed comprehensive bioinformatics and *SlCRT* expression in various tissues and treatments. In the bioinformatics analysis, we described the physiochemical properties, phylogeny, subcellular positions, chromosomal location, promoter analysis, gene structure, motif distribution, protein structure and protein interaction. The phylogenetic analysis classified the CRTs into three groups, consensus with the gene architecture and conserved motif analyses. Protein structure analysis revealed that the calreticulin domain is highly conserved among different tomato species and phylogenetic groups. The cis-acting elements and protein interaction analysis indicate that CRTs are involved in various developmental and stress response mechanisms. The cultivated and wild tomato species exhibited similar gene mapping on chromosomes, and synteny analysis proposed that segmental duplication plays an important role in the evolution of the CRTs family with negative selection pressure. RNA-seq data analysis showed that *SlCRTs* were differentially expressed in different tissues, signifying the role of calreticulin genes in tomato growth and development. qRT-PCR expression profiling showed that all *SlCRTs* except *SlCRT5* were upregulated under PEG (polyethylene glycol) induced drought stress and abscisic acid (ABA) treatment and *SlCRT2* and *SlCRT3* were upregulated under salt stress. Overall, the results of the study provide information for further investigation of the functional characterization of the CRT genes in tomato.

## 1 Introduction

Calreticulins (CRTs) are ubiquitous proteins localized in the eukaryotic cells’ endoplasmic reticulum (ER). These chaperones fold newly synthesized proteins and act as buffering proteins to regulate Ca^2+^ homeostasis (Thelin et al., 2011). The first purified CRT-like Ca^2+^ storage protein in plants was obtained from spinach leaves (Menegazzi et al., 1993). Later many genes encoding CRT as a potential calcium-binding protein were cloned and characterized in various plant species like Arabidopsis (*Arabidopsis thaliana*), barley (*Hordeum vulgare*), Chinese cabbage (*Brassica rapa*), maize (*Zea mays*), tobacco (*Nicotiana tabacum*), wheat (*Triticum aestivum*), castor bean (*Ricinus communis*), rice (*Oryza sativa*) and petunia (*Petunia species*) (Chen et al., 1994; Denecke et al., 1995; Kwiatkowski et al., 1995; Lim et al., 1996; Coughlan et al., 1997; Nelson et al., 1997; Li and Komatsu, 2000; Jia et al., 2008; Lenartowski et al., 2014). Structurally, plant CRT is similar to animal CRT; however, the number of CRT varies among animals and plants. In animals, the CRT family contains two CRT genes (CRT1 and CRT2) (Persson et al., 2002). At the same time, three CRT members exist in plants, classified into two different subgroups, CRT1 and CRT2, designated as one group and CRT3 as another group (Persson et al., 2003; Christensen et al., 2010). The plant CRT proteins mainly comprise 420 amino acids with three major structural domains. The N terminus of the protein contains a highly conserved N domain, categorized by the existence of two calreticulin-specific motifs. The central region contains a rich sequence of proline residues and is designated as the P domain. This protein region has high affinity but low calcium binding capacity. The remaining region towards the C-terminus represents a high calcium binding capacity C domain ending with an ER retention sequence (Matsuoka et al., 1994; Joshi et al., 2019).

The CRT participates in cellular and biological processes, such as cell signaling, protein folding, Ca^2+^ binding and storage, gene expression, ER targeting and retention signals, cell-to-cell communication and plant developmental and stress responses (Jia et al., 2009; Komatsu et al., 2009; An et al., 2011; Thelin et al., 2011). Ca^2+^ deficiency-like symptoms cause yield losses in crops. However, the maize CRT gene enhanced Ca^2+^ accumulation and improved plant nutrient content in co-expressed tobacco and tomato plants (Wu et al., 2012). The overexpression of CRT1 modulates calcium homeostasis and enhances root and shoot regeneration in transgenic plants (Jin et al., 2005). Earlier, it was shown that the petuna *PhCRT* gene is highly expressed in germinating pollen, pistil-transmitting tract cells, fertilization and early embryogenesis (Lenartowski et al., 2014). In sexual reproduction, growing pollen tubes require stabilization of the Ca^+2^ gradient and CRT translation on ER membrane-bound ribosomes controlled Ca^+2^ concentrations within the tube cytoplasm (Suwińska et al., 2015; Suwińska et al., 2017). The high expression of CRT1 regulates Ca^2+^ homeostasis during sexual reproduction and pollen grain formation and maturation (Lenartowski et al., 2015; Wasąg et al., 2017; Suwińska et al., 2022).

CRT plays a key role in plant defense responses and resistance to biotic and abiotic stresses (Chen et al., 2005; Jia et al., 2009; Qiu et al., 2012). In Arabidopsis, salicylic acid level and transcript of genes associated with systemic acquired resistance increased after *Pseudomonas syringae* pv. tomato DC3000 infection. It was found that the increase in endogenous salicylic acid level was due to the CRT2 C-terminus domain calcium buffering activity in response to pathogen attack (Qiu et al., 2012). Another study revealed that CRT3 loss of function resulted in low accumulation of the elongation factor Tu receptor (EFR), an important pattern recognition receptor that performed functions in pathogen-associated molecular pattern-triggered immunity (PTI), and this low accumulation of EFR enhanced the susceptibility of the *crt3* mutant to both virulent and non-virulent strains of pathogen compared to the wild type (Li et al., 2009). The efficient plant-mycorrhiza symbiotic association requires proper Ca^2+^ storage and mobilization. This association is important for plant survival in severe environmental and heavy metal stress conditions (Cheng et al., 2021; Riaz et al., 2021). The CRT was found to be essential for calcium mobilization and aluminum stress response in the mycorrhizal roots of *Medicago truncatula* (Sujkowska-Rybkowska and Znojek, 2018).

In Arabidopsis, CRT1, CRT2 and CRT3 triple knockout mutants increased sensitivity to drought stress, suggesting that CRT is involved in folding proteins related to drought stress in plants (Kim et al., 2013). In wheat, the *TaCRT1* and *TACRT3* expressions were induced under drought stress conditions, and the *TaCRTs* overexpressed lines maintained higher physiological indices related to drought tolerance (Li et al., 2009; An et al., 2011). Similarly, salt stress-induced expression of wheat CRT genes and *TaCRT1* enhanced antioxidant activities to reduce salt stress damage in transgenic tobacco plants (Xiang et al., 2015). In rice, calreticulin interacting protein 1 (CRTintP1) accumulated higher in the cold-tolerant variety compared to the intermediate varieties, and CRTintP1 transgenic lines showed a higher survival percentage in cold stress conditions (Komatsu et al., 2007).

Although several CRT genes are identified in different plant species, however, few analyses of the CRT family in evolution have been conducted. Therefore, it is a pressing need and of great importance to better understand the CRT family members in tomato, because of their essential role in various biological processes and stress responses. Tomato is an important vegetable crop and model research plant with wide consumption and demand worldwide. Due to climate change and diverse growing conditions, tomatoes are exposed to different environmental stresses that harm their development and productivity. In this study, we performed a detailed bioinformatics analysis and comprehensive characterization of the CRT gene family in cultivated and wild tomato species, including physicochemical properties, gene structure, chromosomal location, cis-acting element analysis, intra- and interspecific homology, gene localization and synteny analysis. The tissue-specific expression of *SlCRTs* was performed using digital data. We also investigated the expression pattern of *SlCRTs* under drought, salt, and ABA treatments. The findings of this study will provide the foundation for further detailed functional analysis of the tomato CRT gene family.

## 2 Materials and methods

### 2.1 Sequence retrieval and characterization of CRT genes in tomato

The CRT family members in the four tomato species were identified in the protein sequences retrieved from the Solanaceae Genomics Network (https://solgenomics.net/, accessed November 15, 2023). The HMM profile of the CRT domain (PF00262) was downloaded from Pfam (http://pfam.xfam.org/, accessed November 15, 2023), and searched against downloaded protein sequences by using HMMER search (https://www.ebi.ac.uk/Tools/hmmer/, accessed November 15, 2023). All the predicted CRT family members were further confirmed through hmmscan and SMART database (http://smart.embl.de/, accessed November 17^th^, 2023) for the presence of CRT domains. The CRT protein’s physicochemical parameters, such as amino acid number, molecular weight, theoretical pI, isoelectric point, grand average of hydropathicity (GRAVY) and instability index were computed through the EXPASY ProtParam Tool (http://www.expasy.org/tools/protparam.html, accessed November 19, 2023) and the proteins subcellular localization were predicted with the SubCELlular Localization Predictor Tool (http://cello.life.nctu.edu.tw/, accessed November 19, 2023).

### 2.2 Gene structure, conserved domain and motif identification

The CDS and genomic sequences of CRT family members were used to survey the exon/intron organization and analyze the gene structure by the genes structure display Server program (GSDS) online server (http://gsds.cbi.pku.edu.cn, accessed November 22, 2023). For the evaluation of conserved motifs and domain, the CRT protein sequences were searched using the Multiple Expectation Maximization for Motif Elicitation (MEME) (http://meme-suite./org/tools/meme, accessed November 22, 2023) and HMMER online tools, respectively. Further, the Gene Structure View package in the TBtools toolkit was used for visualization (Chen et al., 2020).

### 2.3 Phylogenetic analysis

In addition to four tomato species, amino acid sequences of five other monocot and dicot plants were used for the phylogenetic analysis (**Supplementary Table S1**). The CRT protein sequences of these species were retrieved using HMMER search. All the protein sequences were aligned with the MUSCLE method and the phylogenetic tree was constructed in Mega 11 using the neighbor-joining method with 1000 bootstrap values. Further, the tree was designed with the ITOL tree online tool (https://itol.embl.de/, accessed November 25, 2023).

### 2.4 Chromosomal location, gene duplication and synteny analysis

The chromosomal position of the 21 candidate CRT genes was retrieved from the genome annotation file and visualized using the Show Genes on Chromosomes- Gene Location Visualize package in TBtools (Chen et al., 2020). The MCScanX toolkit (https://github.com/wyp1125/MCScanX, accessed November 28, 2023) was used to calculate the gene duplication for tomato CRT genes (Wang et al., 2012). The homologous gene duplication and divergence events and the selection pressure on duplicated genes indicated by Ks and Ka, respectively were determined in the TBtools software. The times of duplications in each gene pair were calculated through the formula T = Ks/2λ, where λ= 1.5 x10^-8^ substitutions/synonymous site/year (Blanc and Wolfe, 2004). The degree of homology and evolutionary divergence tree for the CRT genes of the tomato and six other plant species was computed and visualized using the Dual Synteny Plotter implemented package in the TBtools.

### 2.5 Promoter region analysis for cis−acting regulatory elements

To predict the *cis*−acting regulatory elements in the promoter region, sequences of 2000bp (upstream of the transcription start site) were extracted for the CRT members of both cultivated and wild-type tomato species. The acquired sequences were examined in PlantCARE database (https://bioinformatics.psb.ugent.be/webtools/plantcare/html/, accessed November 23, 2023) and the obtained results were visualized and classified.

### 2.6 Protein modeling prediction and protein-protein interactions

The Phyre^2^ online portal (http://www.sbg.bio.ic.ac.uk/phyre2/html, accessed December 09, 2023) at the intensive modeling mode was used to predict the structure of the of CRT proteins in the cultivated tomato (Kelley et al., 2015). To further depict the role of the CRT protein family, the functional interactions between *SlCRTs* were obtained using amino acid sequences for protein-protein in STRING database (https://string-db.org/, accessed December 10, 2023).

### 2.7 RNA sequencing analysis of *SlCRT* genes

Expression profiles for the *SlCRT* genes were carried out from the Tomato Functional Genomics Database (http://ted.bti.cornell.edu/cgi-bin/TFGD/digital/home.cgi, accessed December 15, 2023). The expression patterns of *SlCRTs* were investigated in various tissues including leaf, root, bud, flower and fruit stages (1cm, 2 cm, 3 cm, mature green, breaker, and breaker+ 10). TBTools software was utilized to visualize and create a heat map for analysis of the data.

### 2.8 Plant material, growth conditions and treatments

Tomato (*Solanum lycopersicum*) cv. M82 was used to study the expression patterns of *SlCRT* genes. First, the seeds were washed and soaked with warm distilled water and then placed in water-saturated filter paper for germination. The healthy germinated seeds were transferred to seedling trays containing a mixture of soil, peat, and vermiculite and placed in the growth room under control conditions at 25°C/18°C, 16 h light/8 h dark photoperiod with a relative humidity 60-70%. One-month-old seedlings were exposed to ABA treatment and abiotic stress conditions. In ABA treatment, 100 µM solution was sprayed on seedlings until the solution dripped from the leaves and an equal amount of mock solution was used as a control. The drought and salt stresses were imposed by applying 15% polyethylene glycol (PEG-6000) and 200 mM sodium chloride solutions, respectively. The composite leaf samples were collected from five uniform seedlings at 0, 3, 6, 12 and 24 hours. The samples were immediately frozen in liquid nitrogen and stored at -80 °C for further analysis.

### 2.9 RNA extraction and qRT-PCR analysis

Total RNA Extraction Kit, Polysaccharide Polyphenol Plant (DP441, Tiangen, Beijing, China) was utilized to extract the RNA from the selected samples, following the manufacturer’s instructions. The first strand of cDNA was synthesized using 5 × All-In-One RT MasterMix (G492, ABM, Vancouver, Canada) according to the labeled instructions. NCBI primer design tool (https://www.ncbi.nlm.nih.gov/tools/primer-blast) was used to design *SlCRT* genes qRT-PCR specific primers (**Supplementary Table S2**). The total reaction mixture was 20 µl containing 1 µl of cDNA template, 10 µl of SYBR qPCR Master Mix (Q711, Vazyme, Nanjing, China), 0.5 µl each primer and 8 µl of sterile distilled water. Quantitative RT-PCR was carried out using LightCycler^®^ real-time fluorescent quantitative PCR system (Roche, Basel, Switzerland) with PCR conditions: at 95 °C for 5 min, followed by 40 cycles of 95 °C for 10 s and 60 °C for 30s. The relative expressions of the genes were calculated using the 2^−ΔΔCt^ method (Livak and Schmittgen, 2001) with the *SlActin* gene as a normalized control.

### 2.10 Statistical analysis

SPSS (IBM, Armonk, New York, USA) software was used for data analysis. One-way ANOVA *post hoc* Least Significant Difference (LSD) test was used for multiple comparisons with a level of significance *p* ≤ 0.05 and were expressed as the mean ± standard error (SE) of three replicates.

## 3 Results

### 3.1 Genome-wide analysis reveals consistency of CRT in tomato

The hidden Markov model (HMMER) 3.0 was used to identify the tomato CRT family candidate genes. The predicted amino acid sequences were verified using hmmscan and SMART for the presence of the CRT domain “PF00262”. Finally, 5, 5, 5 and 6 CRT genes were identified in *S. lycopersicum*, *S. pennellii*, *S. pimpinellifolium*, and *S. lycopersicoides*, respectively. The identified genes were named S*lCRT1* to *SlCRT5*, *SpiCRT1* to *SpiCRT5*, *SpCRT1* to *SpCRT5*, and *SlydCRT1* to *SlydCRT6* based on their chromosomal location (**Supplementary Table S3**).

### 3.2 Physiochemical properties of CRT proteins and prediction of localization

Important characteristics of 21 CRT proteins from four tomato species are presented in **Table 1**. In case of *SlCRT*, the CDS length ranged from 1242 (*SlCRT3*) to 1617 (*SlCRT2*), with an average length of about 1400 bp. The length of amino acids ranged from 413 (*SlCRT3*) to 538 (*SlCRT2*), molecular weight in the range of 47. 60 to 48.54, isoelectric point values in the range of 4.50 to 6. 55, an aliphatic index in the range of 56.64 to 72.34 and an instability index between 35. 53 to 44.30. Similarly, the CDS length varied from 645 to 1617 in the other three species and the amino acid length from 214 to 538. Whereas the molecular weight, instability index and aliphatic index in the three wild species varied from 24.97 to 60.97, 34.03 to 43.1 and 56.64 to 98.41. Proteins CRT1 and CRT3 were stable in nature with lower instability index in all four species. The results of isoelectric points indicated that all CRT proteins are acidic with low pi (pI < 7) values except *SpiCRT3* (8.37). Hydrophilicity analysis showed that all 21 CRT proteins are hydrophilic with negative GRAVY values. The subcellular localization prediction of each member of CRT was performed and it was found that CRT proteins were located in the endoplasmic reticulum, nucleus and cytoplasm (**Table 1**). CRT1 and CRT2 proteins of cultivated and wild tomato species were predicted to be located in the endoplasmic reticulum and CRT3 in the nucleus, except *SpiCRT3*, which is in the cytoplasm.

**Table 1:** Detailed information and protein properties of identified calreticulin genes in different tomato species.

Species	Gene name	CDS (bp)	No of AA	MW (kDa)	pl	Aliphatic index	GRAVY	Instability index	Subcellular localization
	*SlCRT1*	1254	417	47.602	4.50	56.64	-0.989	35.53	ER
	*SlCRT2*	1617	538	61.033	4.69	72.34	-0.73	42.66	ER
*S. lycopersicum*	*SlCRT3*	1242	413	48.541	5.97	61.14	-0.992	37.88	Nucl
	*SlCRT4*	1335	444	52.252	6.55	63.04	-1.073	44.3	Nucl
	*SlCRT5*	1599	532	60.837	4.75	72.63	-0.778	42.65	Cyto
	*SpiCRT1*	1173	390	44.606	4.76	60.28	-0.87	36.80	ER
	*SpiCRT2*	1617	538	61.033	4.69	72.34	-0.73	42.66	ER
*S. pimpinellifolium*	*SpiCRT3*	1326	441	51.236	8.37	77.14	-0.534	35.57	Cyto
	*SpiCRT4*	1248	415	47.922	5.25	72.82	-0.705	35.40	Cyto
	*SpiCRT5*	1617	538	61.422	4.79	72.55	-0.773	42.73	Cyto
	*SpCRT1*	1254	417	47.602	4.50	56.64	-0.989	35.53	ER
	*SpCRT2*	1617	538	61.008	4.68	70.54	-0.764	42.82	ER
*S. pennellii*	*SpCRT3*	1242	413	48.557	6.09	62.08	-0.979	37.35	Nucl
	*SpCRT4*	1302	433	51.003	6.12	64.64	-1.038	42.52	Nucl
	*SpCRT5*	1605	534	61.049	4.75	73.45	-0.774	42.47	Cyto
	*SlydCRT1*	1242	413	47.190	4.50	58.14	-0.966	37.99	ER
	*SlydCRT2*	1617	538	60.977	4.68	72.16	-0.728	42.09	ER
*S. lycopersicoides*	*SlydCRT3*	1152	383	44.627	6.38	64.13	-0.915	34.03	Nucl
	*SlydCRT4*	1113	370	43.071	5.77	61.68	-0.991	34.33	Cyto
	*SlydCRT5*	645	214	24.973	5.33	98.41	-0.228	43.19	Extr
	*SlydCRT6*	1617	538	61.355	4.80	73.27	-0.769	42.55	Cyto

Amino acid (AA), molecular weight (MW), isoelectric point (pI), endoplasmic reticulum (ER), nucleus (Nucl), cytoplasm (Cyto) and extracellular space (Extr).

### 3.3 Evolutionary relationship of CRT genes

**Figure 1:**
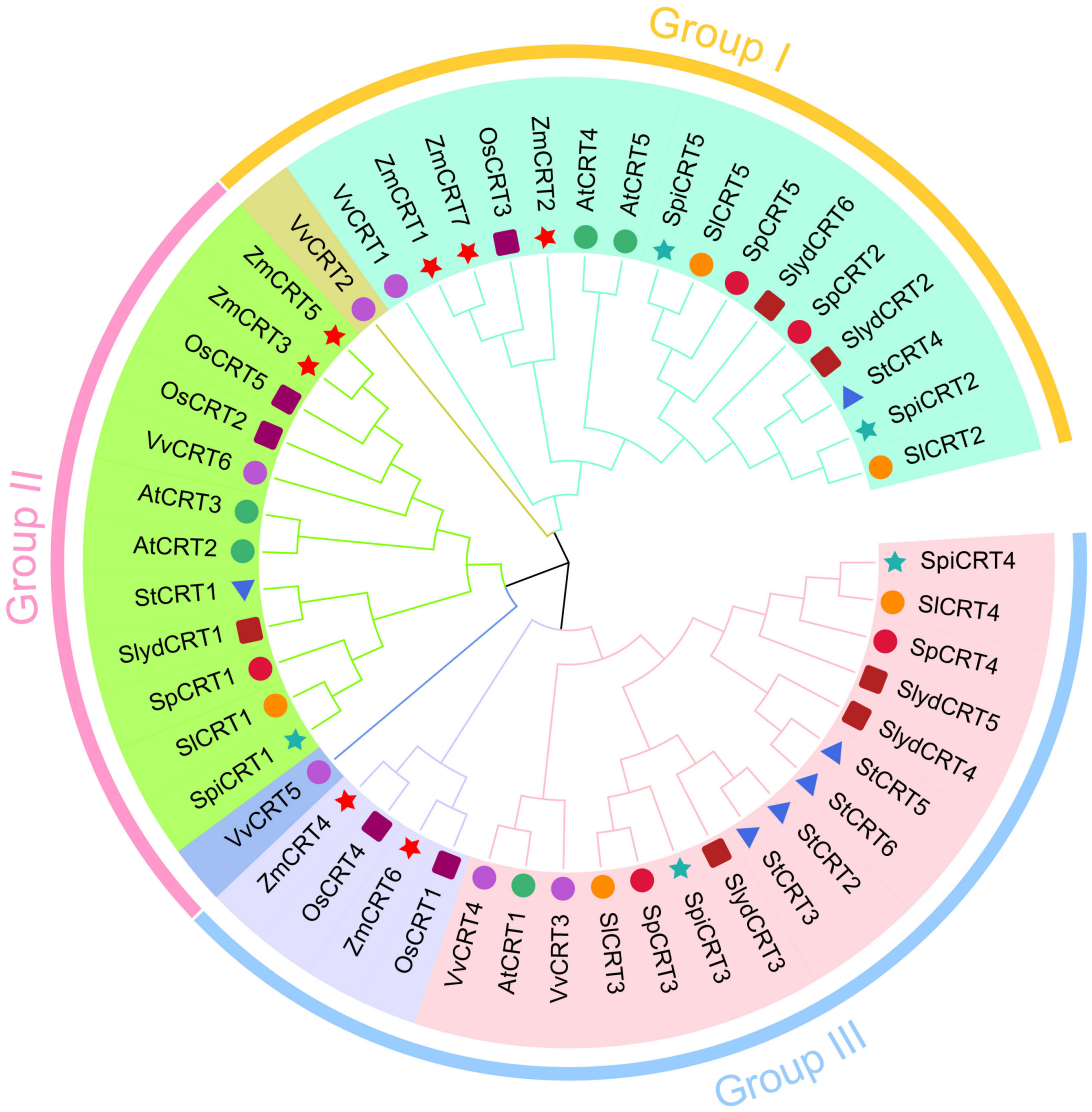
Phylogenetic analysis among CRT members. A total of fifty proteins in nine plant species were identified and used for phylogenetic tree construction. The number of proteins from each species includes; *S. lycopersicum* (5 members), *S. pimpinellifolium* (5 members), *S. pennellii* (5 members), *S. lycopersicoides* (6 members), *A. thaliana* (5 members), *S. tuberosum* (6 members), *Vitis vinifera* (6 members), *O. sativa* (5 members), and *Z. mays* (7 members). The MEGA 11 with MUSCLE method for protein alignments and NJ method (1000 iteration) was used for tree construction. The different colors showed different phylogenetic groups.

To investigate the evolutionary history of the CRT family members, all the CRT protein sequences, 21 from tomato and 29 from other monocot and dicot species were utilized to construct an unrooted phylogenetic tree with the MEGA 11.0 software. Based on the bootstrap and the tree’s topology, the CRT genes were categorized into three groups designated as I, II and III (**Figure 1**). Group III harbored 20 CRTs, represented the biggest and groups I and II contained 17 and 13 CRTs respectively. The CRT members from all selected plant species were distributed in all three groups, among which cultivated tomato *SlCRT2* and *SlCRT5* were distributed in group I, *SlCRT1* in group II and *SlCRT3* and *SlCRT4* in group III. The wild tomato CRT genes showed a similar distribution pattern as the cultivated tomato, except for *S. lycopersicoides* in which *SlydCRT2* and *SlydCRT6* clustered in group I, *SlydCRT1* in group II and *SlydCRT3, SlydCRT4* and *SlydCRT5* in group III. The cultivated tomato *SlCRT* genes are more closely related to the members of the CRT gene families of its wild progenitors and the clustering of different species CRT members into the same group suggested that they have similar origins, evolutionary history and relationships.

### 3.4 Gene structures and motif analyses of the tomato CRT gene family

**Figure 2:**
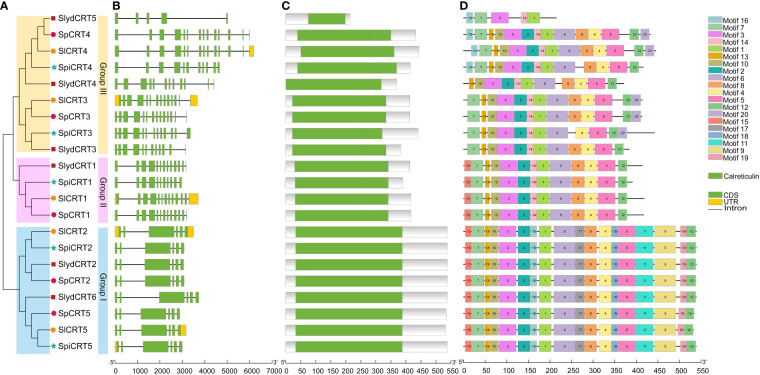
Phylogenetic relationship, genomic architecture, conserved domains & motifs of *CRT* genes in tomato. **(A)** Phylogenetic tree; different shapes and colors represent different species and groups. **(B)** Exon, intron and untranslated regions (UTRs) of tomato CRT genes **(C)** Distribution of conserved domain of CRT. The green boxes represent the conserved calreticulin domain. **(D)** The motif composition of CRT proteins. The 20 different motifs are visualized in different colors.

To study the structural diversity of CRT genes of different tomato species, we compared the intron and exon structures with their corresponding genomic DNA sequences by constructing an unrooted phylogenetic tree of CRT genes (**Figure 2A**). The analysis showed that the CRT gene members in four tomato species were clustered into three groups. Group III was the dominant with 9 members followed by group I (8 genes) and group II had 4 genes. The intron and exon numbers varied in each group, ranging from 4 to 13 and 5 to 14 respectively (**Figure 2B**). Furthermore, members clustered in the same group shared similar exon-intron structures, including the number of exons and introns. For instance, all the genes in group one contained 7 introns and 6 exons. In contrast, CRTs in group III showed diversity in the exon and intron numbers with *SlydCRT5* containing only 5 exons and 4 introns. Protein structure analysis of the CRTs revealed that the length and position of the calreticulin domain were highly conserved among different tomato species and phylogenetic groups (**Figure 2C**). The domain positions in group one, group two and most of the group three members were slightly towards the N-terminal, while in group three the *SlydCRT4* and *SlydCRT5* candidates domains were more towards N-terminal and C-terminal than other members, respectively. Furthermore, the identity percentage of all the CRT protein sequences in the four tomato species was 51.77%, which varied in each group, with 97.72%, 89.87% and 64.1% in I, II and III groups, respectively. To further describe the structural diversity of CRT members from the four tomato species, the analysis of the conserved motif using MEME tool depends on the amino acid sequences with twenty motifs (**Figure 2D**; **Supplementary Table S4**). The detected motifs length ranged from 11 to 50 amino acids and almost all CRT genes studied here contained motifs 1 and motif 3. Further, members within group one and group two shared exactly similar motif numbers and patterns. In both groups, motifs 12 and 15 were distributed at the C-terminus and N-terminus of motif patterns, respectively. However, it was found that the variation in the domain position of the two members (*SlydCRT4* and *SlydCRT5*) changed the motif numbers and patterns in group three. In conclusion, CRT genes from both cultivated and wild types showed a high degree of homology in structure and motif, implying that they may be closely related and have similar functions.

### 3.5 Putative cis-regulatory element analysis in the CRTs gene promoters

**Figure 3:**
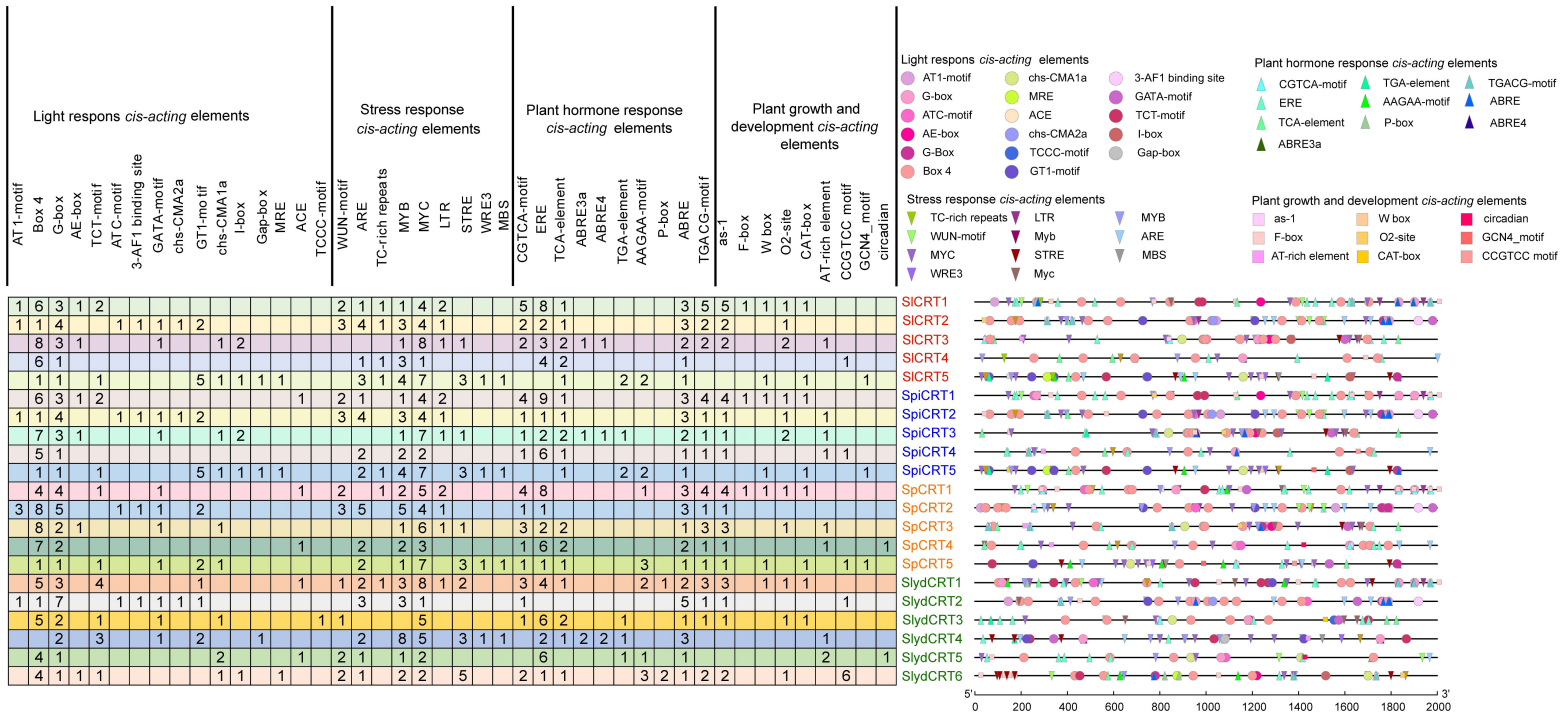
Cis-elements in the promoter regions of *SlCRT, SpiCRT, SpCRT* and *SlydCRT* genes. Division of cis-elements into four major categories according to their function. Each color and shape characterize a specific element and the digit in the box represents the number of each element in the promoter region.

Cis-regulatory elements are essential in the transcriptional initiation of various developmental, hormonal and stress-related genes (Marand et al., 2023). In order to explore the cis-elements in the CRT genes of the four tomato species, 2000 bp upstream promoter regions were retrieved from the Sol Genomics Network. The promoter sequences were then uploaded to the PlantCARE database to identify the putative cis-acting elements. Overall, 47 types of elements were detected in the 21 genes of four tomato species (**Figure 3**). Based on their regulatory and biological functions, the elements were further classified into four categories: light-responsive (17), stress-responsive (11), phytohormone-responsive (10), and plant growth and development cis-elements (9). Two light-responsive cis-elements (Box-4 and G-box), three stress-responsive (ARE, MYC and MYB), three phytohormones responsive (ERE, ABRE, and TGACG-motif), and one plant growth and development cis-element (as-1) were identified in the high ratio in the promoter regions of the different tomato species CRTs genes. Here, the presence of various types and numbers of cis-elements at different positions in the gene promoter region reveals the potential function of the gene.

### 3.6 Chromosome mapping and synteny analysis

**Figure 4:**
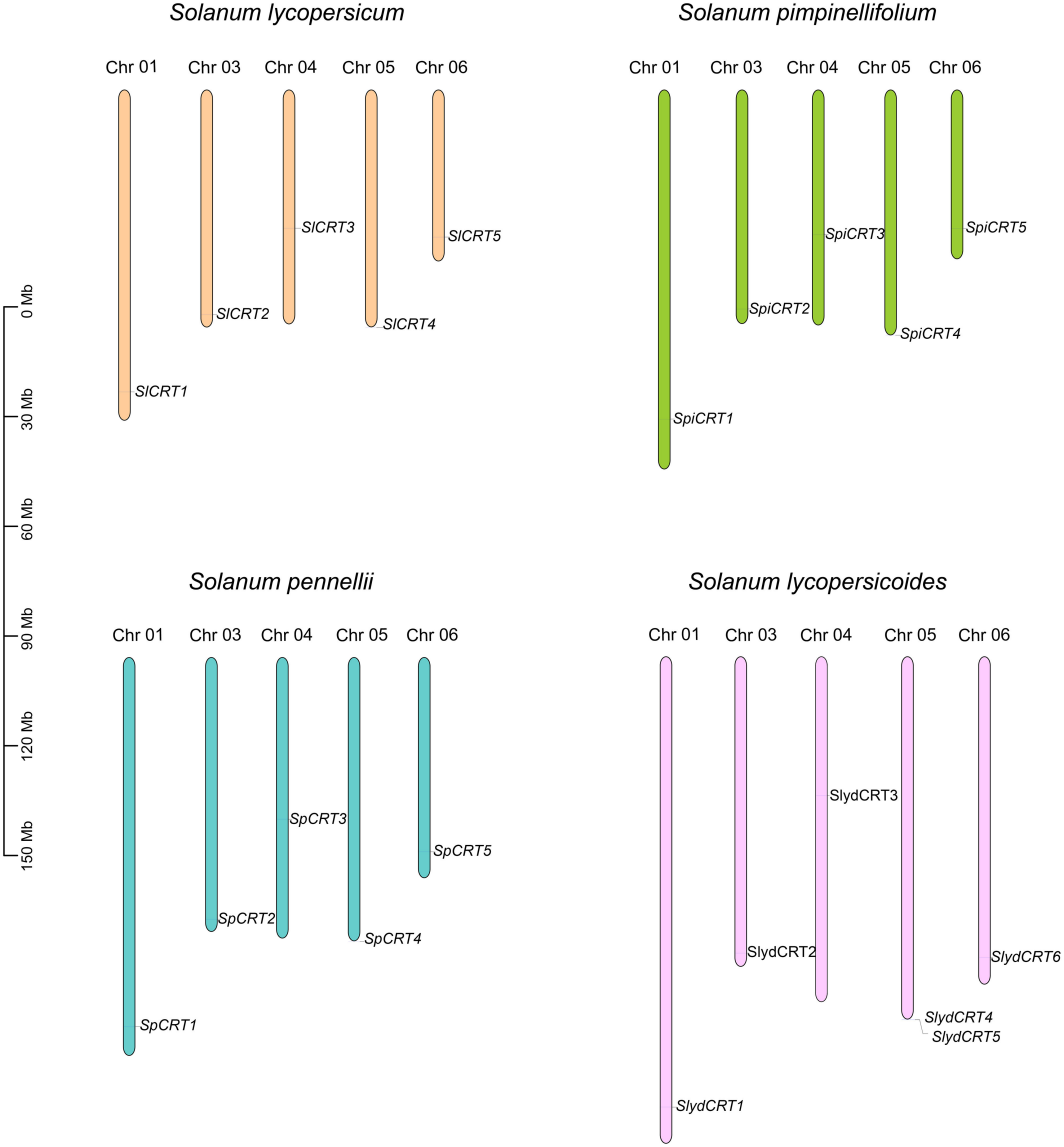
Chromosomal mapping of calreticulin genes on different tomato species. The different color columns and the number displayed on the top of each column represent the chromosomes and chromosome numbers in each species, respectively. The scale is expressed in megabase (Mb).

To examine the chromosomal distribution, each CRT from one cultivated and three wild species was searched and mapped on the respective chromosomes according to the number and position of the chromosome. The results demonstrated that twenty-one CRTs were positioned on six of the twelve tomato chromosomes including chromosomes 1, 3, 4, 5, and 6 (**Figure 4**). Among the species, the distribution of CRT on *S. lycopersicum*, *S. pennellii* and *S. pimpinellifolium* was exactly similar with one gene on each chromosome, while one extra gene of *S. lycopersicoides* mapped on chromosome 5. Almost all the genes of the four species were distributed on one side of the chromosomes except CRT3 which was centrally mapped.

**Figure 5:**
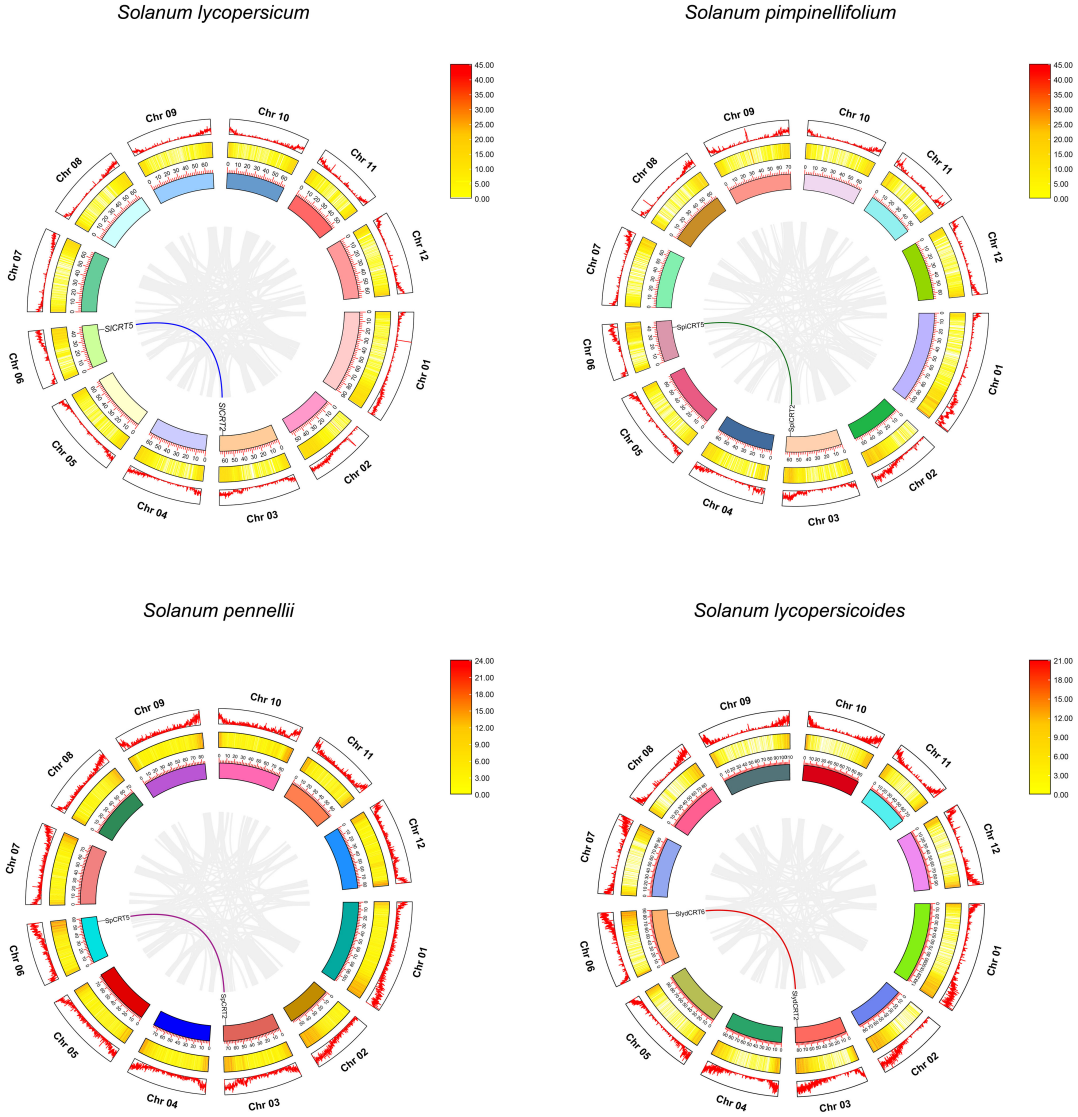
Synteny analysis for CRT gene family in different tomato species. Circle, outside to inside, chromosome number and density (first circle), heat map for gene density on each chromosome (second circle) and ideogram along with coordinates of chromosome (third circle). The different color lines represent the synteny pairs of CRT genes and the gray lines illustrate the synteny of all genes in different tomato species.

Gene duplication events are the key factors involved in the gene amplification and expansion of gene families during the genome’s expansion (Cannon et al., 2004). In this study, we did not find any tandemly duplicated gene pair in the four tomato species, while eight CRTs were found to exhibit four pairs (single pair for each species) of segmental duplication events positioned on the different chromosomes (**Figure 5**). The matching pair genes CRT2-CRT5 were common in *S. lycopersicum*, *S. pimpinellifolium* and *S. pennellii*, while different in *S. lycopersicoides* (CRT2-CRT6). Further, the species evolution assessment revealed that the Ka/Ks ratios for all segmentally duplicated CRTs gene pairs were less than 1, which implies that during the evolution process, tomato CRTs have gone through pure selection (**Supplementary Table S5**). The predicted divergence time showed that duplication of *SlCRT2* & *SlCRT5* in *S. lycopersicum*, *SpiCRT2* & *SpiCRT5* in *S. pimpinellifolium*, *SpCRT2* & *SpCRT5* in *S. pennellii*, and *SlydCRT2* & *SlydCRT6* in *S. lycopersicoides* occurred approximately 24.55, 24.46, 24.78 and 23.52 Mya, respectively. Segmental duplication is suggested to play a crucial role in amplifying the CRT family in tomato species.

**Figure 6:**
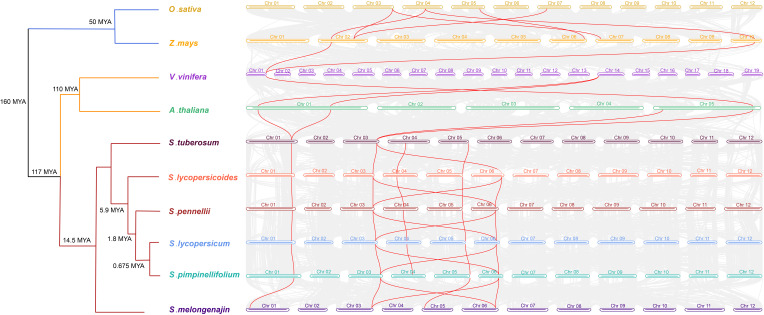
Divergence times and the syntenic relationship between homologous CRT genes of tomato and other plant species. The evolutionary divergence tree is on the left side of the figure with divergence time and name of species in each branch. The horizontal bars of different colors and numbers characterize the chromosomes of different species. The lines in red color represent the synteny pairs of CRTs between different plant species.

Further, interspecies collinearity analysis was performed to identify the homologs of the CRT genes between four tomato species, two monocots (*O. sativa* and *Z. mays*) and four dicots (*A. thaliana, S. melongena, S. tuberosum* and *Vitis vinifera*) species. The results showed that five tomato CRT genes were collinear with *S. tuberosum*, four were collinear with *S. melongena* and only two were with other plant species (**Figure 6**). However, the divergence time revealed higher intraspecific homology among different Solanaceae members and a distant relationship with other plant species. This homology was probably because of their close kinship.

### 3.7 Predicted protein modeling and interaction networks

**Figure 7:**
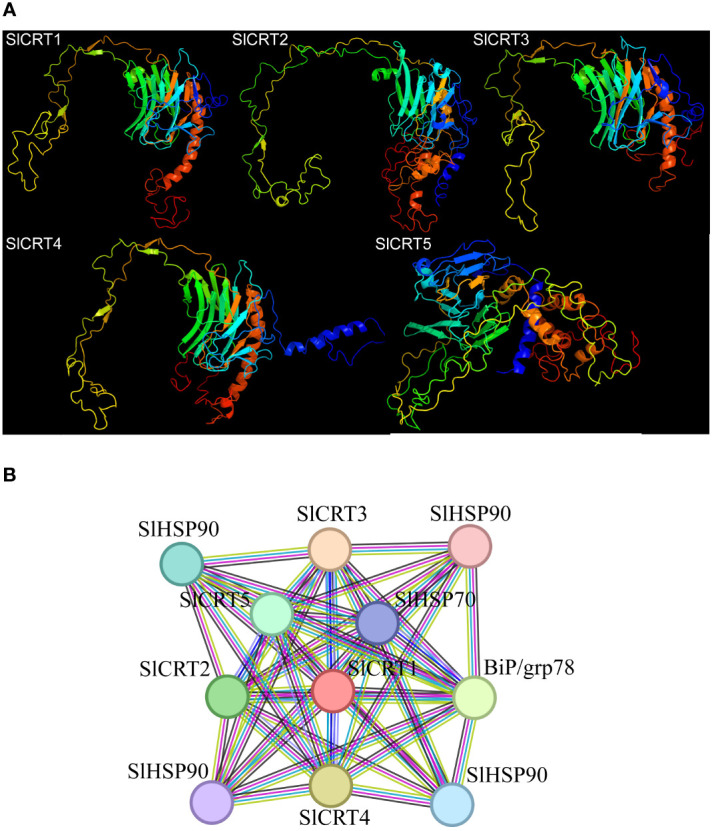
Protein analysis: **(A)** Predicted 3-D structures of CRT proteins. **(B)** Tomato CRT family members interaction network with other proteins.

The five predicted models of the *SlCRTs* were generated using the Phyre2 online tool based on c3rg0A and c1jhnA templates with a 100% probability (**Figure 7A**). The prediction results showed that *SlCRT1*, *SlCRT3* and *SlCRT4* shared similar protein structures, however, *SlCRT4* and *SlCRT5* have more coiled structures compared to the other members. The protein interaction results showed different interactions of *SlCRTs*, where the total number of nodes was 11 with 8.91 average node degree. The STRING analysis revealed 49 edges and three representative local network clusters: CL: 4111, CL: 4113 and CL: 4117. The *SlCRTs* showed interactions with heat shock proteins (Hsps) *SlHSP90* and *SlHSP70* and lumenal binding protein (Bip/GRP78) (**Figure 7B**). Moreover, the protein analysis exhibited a common calreticulin domain (PF00262) in all five members.

### 3.8 Expression of *SlCRTs* in different plant tissues

**Figure 8:**
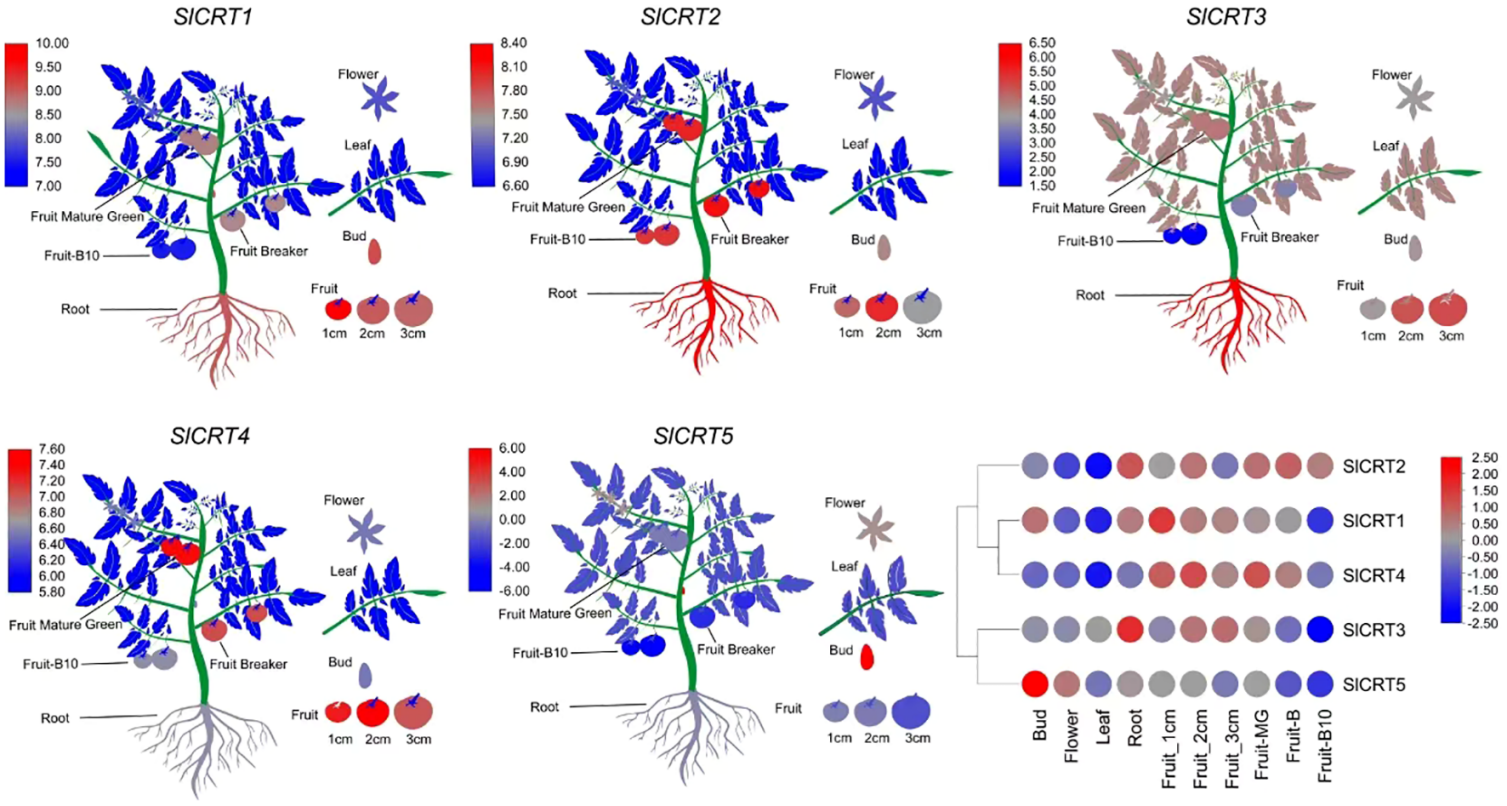
Heatmap of expression profiling of tomato CRT genes in different tissues. RNA-seq data in different tissues of tomato cultivar Heinz (Log2-based RPKM values). Color score from blue to red; low to high expression.

To observe the expression profiles, already published RNA-seq data was used to examine the expression of *SlCRTs* genes in various plant tissues and development stages. The result showed that all five tomato *SlCRT* genes were differentially expressed among the examined tissues (**Figure 8**; **Supplementary Table S6**). *SlCRT1* showed a significantly higher expression level than the other *SlCRTs* in all detected tissues, whereas *SlCRT5* exhibited the lowest transcript accumulation in most tissues except the bud. Furthermore, *SlCRT1*, *SlCRT2* and *SlCRT3* showed maximum expression patterns in roots, buds and different fruit developmental stages, while *SlCRT4* and *SlCRT5* were highly expressed in fruits and buds, respectively. Interestingly, the transcript level of all the CRT genes was lowered in the leaf tissue than in other tissues except for *SlCRT3*, which was expressed at a high level in leaf tissues.

### 3.9 Expression analysis of *SlCRTs* under abiotic and ABA treatments

**Figure 9:**
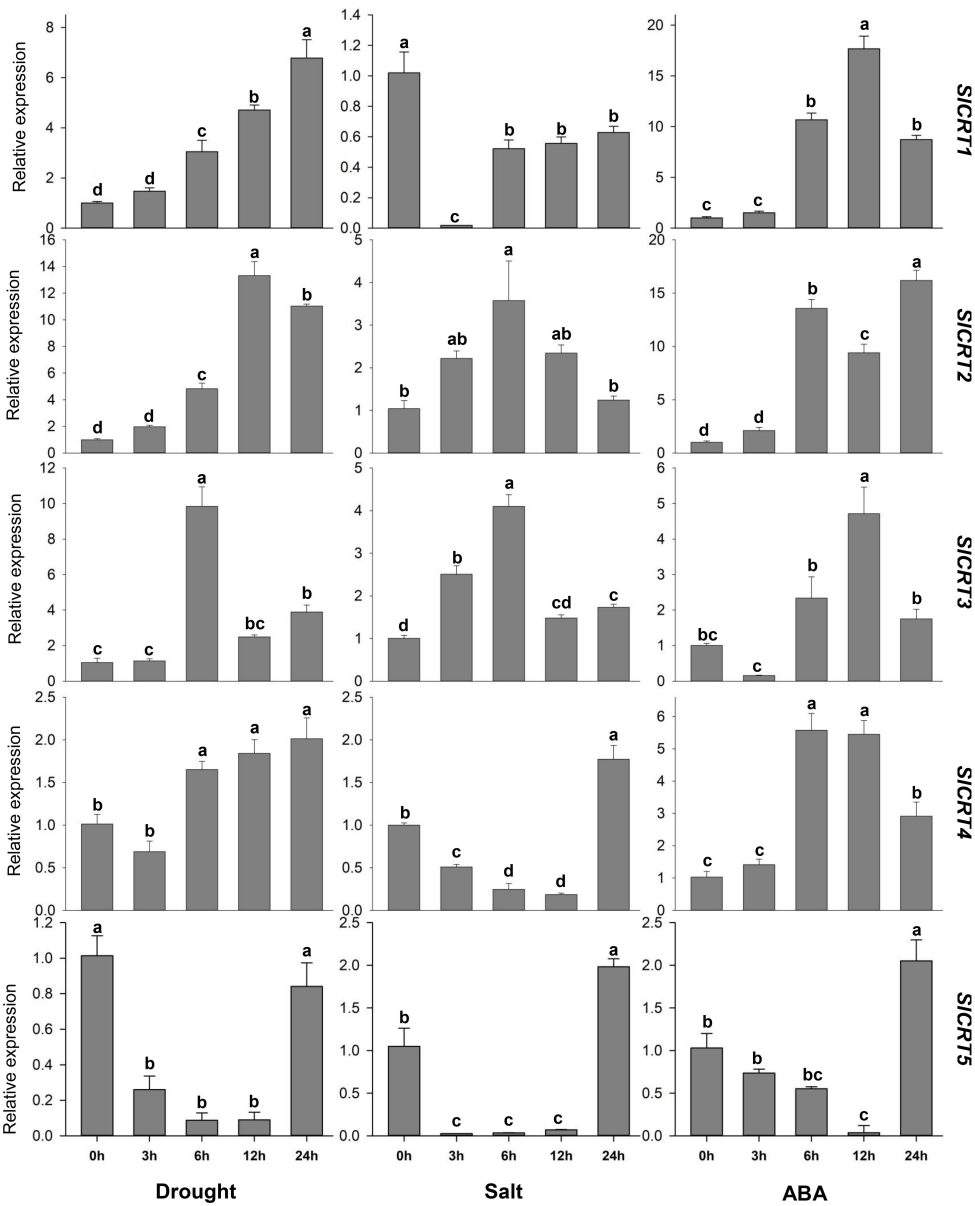
Expression analysis of *SlCRT* genes under drought, salt and ABA treatments at different time points (0, 3, 6, 12 and 24 h). Bars represent the mean values of three replicates ± standard error (SE). Different and the same letters indicate significant and non-significant differences at p ≤ 0.05, respectively.

To determine the expression profiling of tomato CRT genes, quantitative RT-PCR analysis was performed at different time intervals under drought, salt and ABA treatments (**Figure 9**). In drought stress, the expression of *SlCRT1* and *SlCRT2* significantly up-regulated and reached a maximum of 7 and 13 folds at 24 h and 12 h respectively. The *SlCRT3* showed a similar expression to that of control at 1 h, however, increased at 6, 12 and 24 h and peaked 10 folds at 3 h treatment. *SlCRT4* expression plummeted at 1 h but after that steadily increased and rose to 2 folds at 24 h treatment. Interestingly, a gradual decrease was observed in the expression of *SlCRT5* at 3, 6 and 12 h before recovering at 24 h. Under salt stress treatment, the expressions of *SlCRT2* and *SlCRT3* showed similar expression trends as both were initially up-regulated and then fell back. The maximum expression level of *SlCRT2* (3.5 folds) and *SlCRT3* (4 folds) was recorded at 6 h treatment. The expression level of *SlCRT1*, *SlCRT4* and *SlCRT5* significantly downregulated under salt treatment. In SlCRT1, the transcript level was lowered at all treatment time points in comparison to control, however, *SlCRT4* and *SlCRT5* expressions were recovered and induced up to 2 folds at 24 h. In ABA treatment, the expression of *SlCRT1, SlCRT2* and *SlCRT4* exhibited similar expression to that of control at 3 h before reaching a maximum of 18, 16 and 6 folds, at 12, 24 and 6 h, respectively. Additionally, the expression of *SlCRT3* initially downregulated at 3h, however, significantly increased at 6, 12 and 24 h with a maximum expression of 5 folds. The *SlCRT5* showed a gradually low expression level from 3 to 12h time points, but significantly induced (2 folds) at 24 h compared to the control. The differential expression and response to abiotic stresses and ABA treatments implied the potential role of *SlCRT* genes in mediating the tolerance mechanism of tomato.

## 4 Discussion

Calreticulin is a Ca^2+^-binding ER protein that helps fold proteins and performs functions in the maintenance of cellular homeostasis and molecular chaperoning during key developmental processes and stress responses (Wasąg et al., 2017; Hetz and Papa, 2018; Pröbsting et al., 2020). The typical characteristics of CRT proteins are the N-terminus globular domain, the middle proline-rich P domain and the ER-retention sequence domain at the C-terminus with the different functional roles of each domain (Joshi et al., 2019). The calreticulin family has been extensively studied in animals, and described in a few plant species. However, there is a paucity of information about the careticulin genes in solanaceous crops.

Plant CRTs were first described in 1998 and initially classified into two homologous groups (Crofts and Denecke, 1998). In this study, we identified CRT family members in four tomato species and performed comprehensive bioinformatics and expression analysis. The cultivated tomato CRT family contains 5 genes, the same number as that of Arabidopsis, rice, and two wild tomato species but less than that of maize, grapes, potato and *S. lycopersicoides*. The equal number of genes in both cultivated and wild species implies that the cultivated tomato has maintained the CRT gene number during domestication. As continued domestication and selection result in the loss of genes, comparatively wild tomato species characterize a rich gene pool and substantial gene loss makes the cultivated tomato genetic diversity too narrow (Gao et al., 2019; Szymański et al., 2020; Li et al., 2021a). The CRT family is distributed on chromosomes 1, 3, 4, 5 and 6, which was highly consistent in all tomato species except *S. lycopersicoides*, which contained two CRT genes on chromosome 5. Most of the CRT genes were mapped at the same position and towards the terminal ends of the tomato chromosomes. Based on their similar distribution characteristics, the CRT genes showed consistency in their physiochemical properties such as molecular weight, amino acid, isoelectric point, and instability index.

The phylogenetic analysis divided the CRT genes of tomato and other plant species into three groups. Group III contained the most (40%) genes, with one gene from Arabidopsis, two from cultivated and other species, three from *S. lycopersicoides* and four from potato. Previously, the CRT genes of various monocotyledon and dicotyledon species were classified into three subgroups based on their cDNA sequences (Wasąg et al., 2019). Structure analysis of CRT members showed that the phylogenetic groups I and II were more conserved concerning exon/intron numbers or conserved motif, but the group I members had relatively low intron numbers compared to the other two groups, suggesting that group I experienced a significant loss of intron during the evolutionary process. Interestingly, in *S. lycopersicoides* the one extra gene on chromosome 5 (*SlydCRT5*) contained 5 exons and 4 introns, probably a duplicated pseudo gene containing low exons (Persson et al., 2003).

In the analysis of promoter sequences of the CRT genes, we found 47 types of cis-elements in tomato, showing that the CRT family is involved in numerous growth and developmental processes, hormonal regulations, stress responses and other functions. In cis-acting elements, mainly box 4, G-box, MYB, MYC, ERE and ABRE were most significantly enriched in all four species, which proves the involvement of CRT genes in different responses and may affect the life activities of tomato by regulating various biological and molecular processes, which is consistent with previous reports (An et al., 2011; Qiu et al., 2012; Sun et al., 2023).

The gene duplication process is a prevailing feature involved in whole genomes and gene family evolution, consisting of four different events, such as segmental duplication, tandem duplication, whole genome duplication and transposition events (Paterson et al., 2010). However, the prior two duplication mechanisms played a more crucial role in the expansion and evolution of the gene family (Cannon et al., 2004). Previously, it has been demonstrated that both tandem and segmental duplication are involved in the expansion of various tomato gene families, for instance, the NAC and SRO gene families (Jin et al., 2020; Li et al., 2021b). It was observed that only segmental duplication contributed to the evolutionary process of CRT genes in tomato species. Out of five genes, segmental duplication has occurred in one gene pair, with identical genes and chromosomal positions in the cultivated tomato and two wild species (*S. pennellii and S. pimpinellifolium*), however different in *S. lycopersicoides*. The Ka/Ks ratio measures the evolution process as neutral (Ka/Ks=1), positive (Ka/Ks>1) and negative (Ka/Ks<1) (Hurst, 2002). The Ka/Ks ratios of duplicated gene pairs in both cultivated and wild tomatoes were less than 1, and the finding proposed that the duplicated gene pairs were subjected to negative (purifying) selection. Therefore, we hypothesize that the segmental duplications contributed to the expansion of the tomato CRT family, which is a slow-evolving gene family. Previously it was believed that the MYB gene family evolved slowly and that the segmental duplication mechanism occurred in the MYB family (Wang et al., 2015).

Generally, whole genome duplication is an important event in the evolution and expansion of gene family, as it isn’t easy to attain the expansion through single-gene duplication events (Van De Peer et al., 2009). The CRT genes are widespread in living organisms and it indicated that they were initially derived from ancestral gene duplication before the divergence between chlorophyta and embryophyta (Del Bem, 2011). The second round of duplication occurred before the evolutionary split of plants into monocots and dicots, which advocates the existence of CRT orthologs in different plant species (Soltis et al., 1999; Persson et al., 2003). At 14.5 Mya, 117 Mya and 160 Mya tomatoes began to separate with solanaceous crops (potato and eggplant), dicots and monocots, respectively, which shows that the evolution of the CRT genes is slowed. *SlCRT1* and *SlCRT2* are two conserved members of solanaceous crops that maintain a certain degree of identity with ancestral genes. The dicots and monocots shared two homologous CRT pairs, while three to four pairs were between the Solanaceae and other dicots. Similarly, the tomato species shared more syntenic genes with *S. tuberosum* compared to *S. melongena*, the same as among the cultivated and wild tomato species.

The cell requires ER activities to synthesize proteins during growth and under stress conditions. Most secretory and transmembrane proteins mature and fold in the ER, and ER stress repose pathways activate during stress conditions to induce the expression and splicing of transcription factors (Kaur and Kandoth, 2021). The prediction results for SlCRT protein interaction showed that CRT proteins interacted with various transcription factors such as heat shock proteins (Hsps) *SlHSP90* and *SlHSP70* and lumenal binding proteins (Bip/GRP78). Heat shock proteins are molecular chaperones essential to diverse processes such as heat shock transcription factors (Hsfs) regulation, protein folding, signal transduction, growth and development, linked to hormonal responses and biotic and abiotic stress responses (Hahn et al., 2011; Xu et al., 2012; Yadav et al., 2021; Pascual et al., 2023). Similarly, plant BiPs genes are involved in regulating various physiological and molecular mechanisms such as the accumulation of solutes, restoration of alpha-amylase enzyme, stomatal conductance and stress tolerance (Leborgne-Castel et al., 1999; Alvim et al., 2001; Yan et al., 2011; Coutinho et al., 2019; Aghaie and Tafreshi, 2020; Herath et al., 2020). Interestingly, the BiP4 and CRT3 also formed a complex during virus infection and facilitated the movement of viruses in plants (Huang et al., 2023). The interaction of *SlCRTs* with the described proteins indeed enhances the tomato plant ability to regulate function and resist adverse environmental conditions.

Expression pattern of genes in plant tissues can explore important information about their characterization and functional role in different processes. The digital data analysis showed that the three genes *SlCRT1, SlCRT2* and *SlCRT3* specifically expressed in roots, which may be related to root development or growth regulation. *SlCRT4* was highly expressed in all fruit stages from the young to the breaker stage, indicating that it may be involved in early fruit development. *SlCRT5* has higher expression in the flower and bud stages than in the other detected tissues. This gene may be related to the regulation of tomato flowering or the transition to the reproductive stage from the vegetative stage. Previous findings justify our observations, as *PhCRTs* have been well-studied in petunia for their critical role in key reproductive events (Suwińska et al., 2022). In *Brassica rapa*, the *BrCRT1* gene has a role in Ca^2+^ signaling mechanisms related to regeneration and improved shoot and root initiation and regeneration in transgenic tobacco plants (Jin et al., 2005).

Abiotic stress conditions are the major limiting factors for normal plant growth and development. The increased in frequency of extreme weather and changes in climatic conditions exacerbated the adverse effects of the abiotic stresses (Dietz et al., 2021; Razzaq et al., 2021). The primary signals caused by drought and salt stresses are osmotic stress and their secondary effects include oxidative damage. Osmotic stress agents cause an increase in the cytosolic free calcium concentration in plants (Zhu, 2016). As an important inner cellular Ca^2+^ balance regulator, calreticulin is differentially expressed by diverse stress conditions (An et al., 2011). In our study, the qRT-PCR analysis revealed that both drought and salt stress significantly enhanced the transcript level of *SlCRT2* and *SlCRT3*. However, the expression of *SlCRT1* and *SlCRT4* significantly increased and decreased under drought and salt stress, respectively. *TaCRT1*, *TaCRT2* and *TaCRT3-1* genes were previously reported to be strongly induced by salt stress in wheat and *TaCRT1* overexpression improved tolerance against salt stress (Xiang et al., 2015). Similarly, PEG-induced drought stress significantly enhanced the expression of CRT3 in wheat seedlings (Jia et al., 2008). However, it was also reported that the expression of some calreticulin genes was downregulated by stress treatment (An et al., 2011), and these findings were consistent with our current study results. The abiotic stress-induced hyperosmotic signal regulates ABA accumulation, which stimulates various adaptive responses in plants, such as stomatal closure, water potential balance and osmotic pressure balance of cells (Yang et al., 2022). As inferred from the qRT-PCR data, the expression of four *SlCRTs* significantly increased under ABA treatment at different time intervals, whereas the expression of *SlCRT5* was initially downregulated and recovered after 24 h. Here, our cis-element analysis revealed that the *SlCRT1* and *SlCRT2* genes harbor a relatively higher number of phytohormone cis-elements in their promoter regions, which resulted in much higher expression under ABA treatment. Previous studies have reported that exogenous application of ABA upregulated CRT gene expression in *B. napus* young seedlings, and the ABA-induced salt tolerance is regulated by the CRT expression in *Solanum tuberosum* (Shaterian et al., 2005; Liu and Li, 2013). These results indicate the involvement of CRTs in diverse mechanisms related to plant development and abiotic stress tolerance.

## 5 Conclusion

In the present study, for the first time, a detailed integrated analysis including gene identification, physical and chemical properties, gene architecture, chromosomal location, cis-elements, protein interactions, abiotic stress, and ABA-induced specific expression pattern of the tomato CRT genes was performed. 21 CRT members were identified in cultivated tomato and their wild relatives. These genes were clustered in three groups and mapped in similar patterns on the chromosomes of different species. Previously published RNA-Seq data analysis reveals that CRT genes may be involved in tomato growth and developmental changes. qRT-PCR-based expression profiles showed that *SlCRT2* and *SlCRT3* significantly changed under drought, salt, and ABA treatment, which verified their role in tomato abiotic stress tolerance. Overall, these data are helpful for further investigation and provide research directions for gene functional verification studies to identify the role of CRTs in tomato abiotic stress tolerance.

## Data availability statement

The original contributions presented in the study are included in the article/**Supplementary Material**. Further inquiries can be directed to the corresponding authors.

## Author contributions

TM: Conceptualization, Methodology, Writing – original draft. TY: Writing – original draft. BW: Formal analysis, Software, Writing – review & editing. HY: Formal analysis, Methodology, Writing – original draft. DT: Data curation, Software, Writing – original draft. JW: Conceptualization, Resources, Writing – review & editing. QY: Project administration, Resources, Writing – review & editing.
