# Supplementary material for: Comprehensive genomic characterization and expression analysis of calreticulin gene family in tomato
Source: Front Plant Sci. 2024 Apr 22;15:1397765. doi: 10.3389/fpls.2024.1397765 (PMC11070585; doi:10.3389/fpls.2024.1397765)
Supplement: Supplementary file 2 [file Table_2.docx]

**Table S2. The real-time PCR primers used in this study**

| **Gene ID** | **Forward primer(5' to 3')** | **Reverse primer(5' to 3')** |
| --- | --- | --- |
| *SlCRT1* | GTTGGGAAAGCAGGTGGGTA | TCATTGGCGTCACCATTCCA |
| *SlCRT2* | CAAGGGAAAATGGCACGCTC | ATGGCTGCGATAGGCTCAAA |
| *SlCRT3* | GGTATGTGGAAGGCACCCAA | AGAACCTGCCTTTACCTGCC |
| *SlCRT4* | ACATACATGGTGTGGACGGG | ACACTCAAACTTGGCCTCCC |
| *SlCRT5* | AGCGGAATCGGAGAATGTGG | CATGTCCCTCACTCTTGGCA |
| Actin | AGCAGGAACTTGAAACCGCT | CTCATGGATACCCGCAGCTT |
