# Supplementary material for: Comprehensive genomic characterization and expression analysis of calreticulin gene family in tomato
Source: Front Plant Sci. 2024 Apr 22;15:1397765. doi: 10.3389/fpls.2024.1397765 (PMC11070585; doi:10.3389/fpls.2024.1397765)
Supplement: Supplementary file 4 [file Table_4.docx]

**Table S4. Analysis of the 20 conserved motifs of CRT genes in tomato species.**

| **Motif** | **Width** | **Motif sequences** |
| --- | --- | --- |
| 1 | 29 | CETDKLTHVYTFILRPDASYSILIDGREK |
| 2 | 29 | QKKFGGDTPYSIMFGPDICGATTKKLHVJ |
| 3 | 41 | SAKIPEFSNKBGTLVLQYSVKLZQDJECGGGYIKLLSGYVN |
| 4 | 29 | KGEWKRKKIKNPNYKGKWKAPLIDNPEFK |
| 5 | 41 | PDLYVFEPIKYVGIEIWQVKAGSLFDNILIADDPDYAKSVI |
| 6 | 50 | SGSMYTDWDJJPPRKIKDPNAKKPEDWDDREYIEDPDDKKPEGYDEIPPE |
| 7 | 29 | LFVLVLYAVSEVFFEESFDDGWDSRWVKS |
| 8 | 29 | DDPEAKKPEDWDDEEDGEWEAPKIPNPKY |
| 9 | 50 | PFLGEHKSKVLDLLEKAEKQPNJTVGVVISIIIIIFSVLLKLIFGGTKQQ |
| 10 | 21 | WSGDPDDKGJQTSEDAKKYAI |
| 11 | 41 | KTAWKPKFDIEKEKQKAEEEAESGGLKGYQKMVLDLLYKVA |
| 12 | 21 | ZKEEEQEAREEAERRRTEREN |
| 13 | 11 | SEGKAGVWKHT |
| 14 | 15 | LSYQGQNYPIKKELE |
| 15 | 15 | NRRMWSQYALLLLAV |
| 16 | 15 | KHKNPKSGEYIEHHL |
| 17 | 21 | EIEDEEAEKPEGWLDDEPEEI |
| 18 | 15 | GIWKPRDIPNPBYFE |
| 19 | 15 | TDGAETSSSNKZGAE |
| 20 | 21 | DRHRDRYKRRYRRDYDYDDHD |
